# A von Hámos spectrometer for diamond anvil cell experiments at the High Energy Density Instrument of the European X-ray Free-Electron Laser

**DOI:** 10.1107/S1600577523003041

**Published:** 2023-05-09

**Authors:** Johannes M. Kaa, Zuzana Konôpková, Thomas R. Preston, Valerio Cerantola, Christoph J. Sahle, Mirko Förster, Christian Albers, Lélia Libon, Robin Sakrowski, Lennart Wollenweber, Khachiwan Buakor, Anand Dwivedi, Mikhail Mishchenko, Motoaki Nakatsutsumi, Christian Plückthun, Jan-Patrick Schwinkendorf, Georg Spiekermann, Nicola Thiering, Sylvain Petitgirard, Metin Tolan, Max Wilke, Ulf Zastrau, Karen Appel, Christian Sternemann

**Affiliations:** a Technische Universität Dortmund, Fakultät Physik/DELTA, Maria-Goeppert-Mayer-Straße 2, 44227 Dortmund, Germany; b European XFEL, Holzkoppel 4, 22869 Schenefeld, Germany; c Department of Earth and Environmental Sciences, University of Milano-Bicocca, Piazza della Scienza 4, 20126 Milano, Italy; d European Synchrotron Radiation Facility, 71 Avenue des Martyrs, 38000 Grenoble, France; e Universität Potsdam, Am Neuen Palais 10, 14469 Potsdam, Germany; f Universität Rostock, Institut für Physik, Albert-Einstein-Straße 23–24, 18059 Rostock, Germany; g ETH Zürich, Rämistrasse 101, 8092 Zürich, Switzerland; h Universität Göttingen, Wilhelmsplatz 1, 37073 Göttingen, Germany; ESRF – The European Synchrotron, France

**Keywords:** X-ray spectroscopy, X-ray diffraction, inelastic X-ray scattering, diamond anvil cell, spectrometers, imaging spectroscopy, X-ray detectors

## Abstract

A von Hámos spectrometer has been implemented in the vacuum interaction chamber 1 of the High Energy Density instrument at the European X-ray Free-Electron Laser facility. This setup is dedicated, but not necessarily limited, to X-ray spectroscopy measurements of samples exposed to static compression using a diamond anvil cell. Si and Ge analyser crystals with different orientations are available for this setup, covering the hard X-ray energy regime with a sub-eV energy resolution. The setup was commissioned by measuring various emission spectra of free-standing metal foils and oxide samples in the energy range between 6 and 11 keV as well as low momentum-transfer inelastic X-ray scattering from a diamond sample. Its capabilities to study samples at extreme pressures and temperatures have been demonstrated by measuring the electronic spin-state changes of (Fe_0.5_Mg_0.5_)O, contained in a diamond anvil cell and pressurized to 100 GPa, via monitoring the Fe *K*β fluorescence with a set of four Si(531) analyser crystals at close to melting temperatures. The efficiency and signal-to-noise ratio of the spectrometer enables valence-to-core emission signals to be studied and single pulse X-ray emission from samples in a diamond anvil cell to be measured, opening new perspectives for spectroscopy in extreme conditions research.

## Abstract 2

The implementation of a von Hámos spectrometer for diamond anvil cell experiments at the High Energy Density Instrument of the EuXFEL is described.

## 1. Introduction

The application of energy-resolved hard X-ray spectroscopy measurements from samples contained in a diamond anvil cell (DAC) is a well established method at synchrotron facilities. It can be applied to high pressures (*p*) at ambient temperatures (*T*) (see, for example, Badro *et al.*, 1999 ▸; Pascarelli *et al.*, 2004 ▸; Kantor *et al.*, 2006 ▸; Rueff & Shukla, 2010 ▸; Spiekermann *et al.*, 2019 ▸) and when combined with a heating/cooling source at high/low *T* (see, for example, Lin *et al.*, 2007 ▸; Bi *et al.*, 2015 ▸; Rossi *et al.*, 2019 ▸; Wang *et al.*, 2019 ▸; Weis *et al.*, 2019 ▸). The application of X-ray emission spectroscopy (XES) to matter at extreme conditions provides the unique opportunity to study, for example, the electronic spin-state and structure and thus related physical and chemical properties of samples at *p*/*T* conditions expected in the interior of terrestrial and even extraterrestrial planets. The recently implemented novel approach of combining a DAC sample environment with a highly intense pulsed X-ray beam provided by an X-ray free-electron laser (XFEL) facility, such as the European XFEL (Decking *et al.*, 2020 ▸), allows X-ray heating of samples above melting temperatures by accumulative energy deposition caused by volumetric absorption of the 4.5 MHz X-ray pulses, while at the same time measuring changes in the structure of the sample via X-ray analytical techniques (Meza-Galvez *et al.*, 2020 ▸; Liermann *et al.*, 2021 ▸). The X-ray heating provides a more robust heating source due to the volumetric absorption compared with laser heating, where large axial gradients may exist and coupling to the sample may become uneven. Moreover, the heating time during the X-ray heating is reduced to the times of signal collection and so avoids long continuous exposure to continuous-wave lasers, which often promotes chemical migration and reactivity (Sinmyo & Hirose, 2010 ▸).

A first proof-of-principle experiment showed that the combination of X-ray heating via XFEL radiation with a DAC successfully determined the structure and electronic spin-state using a combination of XES and X-ray diffraction (XRD) measurements (Kaa *et al.*, 2022 ▸). To further exploit this approach, we commissioned a hard X-ray wavelength-dispersive von Hámos type spectrometer comprising four analyser crystals in a single column, dedicated but not limited to measurements in DACs. The spectrometer is positioned in the vacuum environment of the interaction chamber 1 (IC1) at the High Energy Density (HED) instrument of the European XFEL. By using a self-amplified spontaneous emission (SASE) beam, this setup is suitable for XES measurements, including the valence-to-core (vtc) signal. In combination with a high-resolution monochromator (Dong *et al.*, 2016 ▸; Wollenweber *et al.*, 2021 ▸) or a seeded beam (Zastrau *et al.*, 2021 ▸), there is great potential for resonant X-ray emission and non-resonant inelastic X-ray scattering (NRIXS) measurements of valence and core electron excitations (X-ray Raman scattering) on a broad energy range in the hard X-ray regime from samples contained in a DAC or from free-standing samples. Thus, the setup extends the list and capabilities of already established hard X-ray von Hámos spectrometers at XFEL facilities (*e.g.* Alonso-Mori *et al.*, 2012 ▸; Szlachetko *et al.*, 2012 ▸; Anwar *et al.*, 2019 ▸; Preston *et al.*, 2020 ▸).

## 2. Spectrometer concept and design

**Figure 1:**
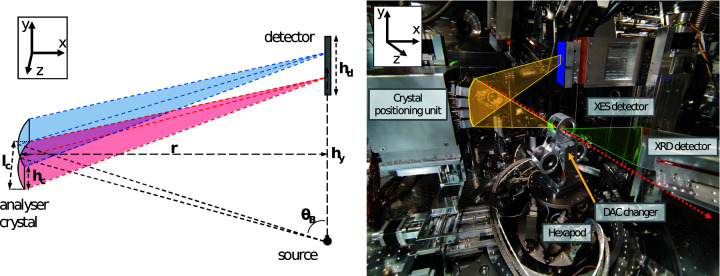
(Left) Scheme of the von Hámos setup. The sample is a point source (black point) that emits at multiple energies, seen from the side for a horizontal scattering angle of 90° relative to the incoming X-ray beam. The detector plane at height *h*_*y*_ has a perpendicular distance *r* to the cylindrically bent analyser crystal with a bending radius of *r* and an area of *h*_c_ × *l*_c_. High-energy (blue) and low-energy (red) X-rays are diffracted with different Bragg angles θ_B_ and focused in the non-dispersive direction leading to a straight line signal in the dispersive direction (*y*) on the detector plane with the size *h*_d_. X-rays propagate along *z* through the emitting source point. (Right) The setup in IC1 includes a DAC exchanger and XRD detectors downstream of the sample. The X-ray beam (red), XES (yellow) and XRD (green) paths are shown. The XES detector array is marked in blue. Example figures of the detector images for different analyser crystal arrangements are shown in Fig. S2.

**Figure 2:**
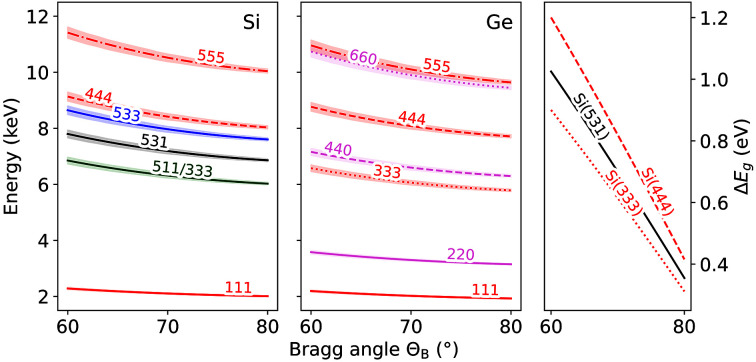
Calculated energy windows for the analyser crystals relative to the Bragg angle for available crystal cuts [Si (left), Ge (centre)] with different reflection orders based on equation (1). Shaded areas show the maximum energy window that can be covered with each crystal. Crystal cuts and their associated *d*-spacings are listed in Table 1 ▸. (Right) Theoretical geometric energy resolution Δ*E*_*g*_ [equation (2)] for a single-crystal spectrometer for cuts Si(531), Si(333) and S(444) on a Jungfrau detector for a vertical beam size of 10 µm and a sample thickness of 10 µm.

For this spectrometer, we used cylindrically bent analyser crystals in a geometrical arrangement first described by von Hámos (1932 ▸). Its schematic is shown in Fig. 1 ▸. X-rays with energy *E* emitted from the sample are diffracted on the 2D detector by the analyser crystal along the dispersive axis following the Bragg equation, 

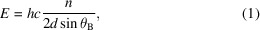

using Planck’s constant *h*, the speed of light *c*, order *n*, interplanar spacing *d* of the analyser crystal, and the Bragg angle θ_B_. The detector plane at height *h*_*y*_ and the sample are situated on the same vertical axis. If only one analyser crystal is used, the crystal with the dimensions *h*_c_ × *l*_c_ is located at half of the height of the detector with a distance equal to its bending radius *r* perpendicular to the sample–detector plane. Thus, each X-ray wavelength is focused along the non-dispersive axis (*z*) to a point on the detector plane, resulting in a line-shaped spectrum along the dispersive direction (*y* direction on the detector plane). To correct for the position offset when using multiple analyser crystals on top of each other parallel to *y*, *r* needs to be slightly decreased and the crystal plane tilted relative to *y*, to achieve focusing on the detector plane and to adopt the correct Bragg angles. The requirements for the spectrometer setup lay in combining high efficiency, *i.e.* a high photon detection yield, and an energy resolution in the sub-eV regime at an adjustable and broad energy range. The positioning of the crystals relative to the incoming beam must be variable to ensure flexible arrangements for different types of measurements in either reflection, transmission or in 90° scattering geometry. The setup is placed in a high vacuum in IC1 (1 × 10^−4^ mbar) on a circular rail surrounding a hexapod sample positioning system (Fig. 1 ▸) to enable a variable 360° scattering angle in the horizontal plane. The four-crystal spectrometer and the X-ray detector are each placed on a combination of three high-precision OWIS linear stages, which are fixed to a relative 180° angle on the circular rail. The linear stages allow for positional alignment of the stack of four crystals relative to the sample position and enable a Bragg-angle range between 60° and 80°, the higher end limited by collision with the sample environment. We use a 2D Jungfrau detector with a pixel size of 75 µm and a sensor array size of 1024 pixel × 512 pixel (Mozzanica *et al.*, 2014 ▸) collecting the reflection from each analyser crystal separately (see Fig. S2 of the supporting information), before adding them together after energy calibration of each spectrum. The alignment of each crystal is carried out independently by a set of three stepper motors controlling the pitch, yaw and roll, which were provided by JJ X-ray (https://www.jjxray.dk/). We use cylindrically bent crystals with various *d*-spacings and with a bending radius of 250 mm and dimensions of 20 mm × 110 mm (V × H). Available crystal orientations and their corresponding *d*-spacings are summarized in Table 1 ▸. The achievable energy ranges for the available analyser crystals are plotted in Fig. 2 ▸. The spectrometer can also be equipped with a combination of different analyser crystals, enabling two or even multi-colour emission measurements. The sample, whether free-standing or confined in a DAC, is aligned to the X-ray beam by a hexapod motion control system. The hexapod can be equipped to carry a DAC exchange system, in the form of three rotating arms, which facilitates the DAC sample turnaround without the necessity to vent/pump the vacuum chamber (Fig. 1 ▸, right). The DAC changer is designed for BX90-RD DACs with a diameter of 50 mm and is moved by a piezo-driven rotational SmarAct GmbH stage. The DAC changer allows for a sample exchange within minutes in contrary to the roughly 60–90 min it takes for the pump–vent procedure of IC1. An in-line microscope with a 10× objective and an extra-long working distance of 35 mm was used for sample visualization and positioning into the beam. The objective had a drilled hole with a diameter larger than the beam along the optical axis so that the X-ray beam can pass during sample alignment, which allowed for simultaneous optical observation. With an optical path, the image was transported outside the vacuum chamber to a Basler2k line-scan camera. The setup allows for the placement of additional detectors in transmission geometry, thus allowing for simultaneously collected XRD measurements, yielding additional information about the sample’s structure.

The spectrometers’ energy resolution mostly depends on the contribution of the source size Δ*E*_*y*_ and Δ*E*_*r*_, as well as the contribution of the pixel size of the detector Δ*E*_*d*_ (Sahle *et al.*, 2023 ▸). The geometric energy Δ*E*_*g*_ resolution can be described by the following equations, 

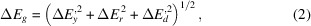

with 

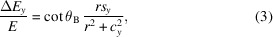




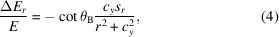




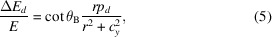

using the vertical beam size *s*_*y*_, the effective horizontal deviation from a point source (including the beam width and sample thickness) *s*_*r*_, the *y* position of the analyser crystal *c*_*y*_, and the detector pixel size *p*_*d*_. For a measurement at a horizontal scattering angle of 90° using the Jungfrau detector at 7050 keV with Si(531) crystals for a beam of size *s*_*y*_ = 10 µm and *s*_*r*_ = 10 µm, the geometric energy resolution is calculated to be Δ*E*_*g*_ = 0.7 eV. We neglected the source size perpendicular to *r*, which can cause a signal shift on the detector along the non-dispersive axis. Further contributions from the Darwin width Δ*E*_DW_ can be determined by the full width at half-maximum (FWHM) of the rocking curve in Fig. S6 (see Table 3). Δ*E*_DW_ at 70° has a value below 0.25 eV for Si(531), Si(333) and Si(444) and thus has only a minor contribution to the energy resolution. Contributions due to compression stress in the bent crystals lattices are not accounted for.

## 3. XES commissioning measurements

The setup was commissioned in the vacuum chamber IC1 at the HED instrument at the European XFEL (Zastrau *et al.*, 2021 ▸). The vacuum was set to a pressure below 1 × 10^−4^ mbar. The SASE beam was operated at a central photon energy of 13.16 keV with an energy bandwidth of Δ*E*/*E* ≃ 3 × 10^−3^ using either single-bunch mode, providing photon pulses at 10 Hz, or 50–200 pulses per train at 455 kHz, with a pulse energy of 800 µJ maximum, *i.e.* 3.8 × 10^11^ photons pulse^−1^ at full beamline transmission. The beam was focused to 60 µm FWHM using Be compound refractive lenses (CRLs) located 9 m upstream of the sample and the size was estimated by measuring the FWHM of the derivative of an edge scan using a tungsten rod. We utilized a set of four Si cylindrically bent analyser crystals either with cut Si(111) or Si(531). The X-ray emission was measured in a 100° horizontal back-scattering geometry, while the Bragg angle on the analyser crystals was controlled by the height of the spectrometer and the detector (Fig. 2 ▸). A Jungfrau detector with a pixel size of 75 µm was used to measure the emission lines at 10 Hz. Simultaneously, we collected the XRD signal with an ePix100 detector (Blaj *et al.*, 2016 ▸) in transmission geometry.

Ambient-pressure XES measurements (Konopkova & Zastrau, 2021 ▸) were conducted on 99.99% pure Fe, Co and Ni foils with a thickness of 5 µm each and GeO_2_ powder compressed between Kapton polymide foils. The 2D XES spectra on the detector were integrated over a region of interest (ROI) along the non-dispersive axis and background corrected by subtracting a ROI at a slightly shifted position on the detector in the non-dispersive direction. The resulting line was energy calibrated by fitting highly accurate reference spectra measured by Hölzer *et al.* (1997 ▸) and Ito *et al.* (2018 ▸) to the *K*α_1_ and *K*α_2_ and the *K*β_1,3_ and *K*β_5_ peak positions, respectively.

**Figure 3:**
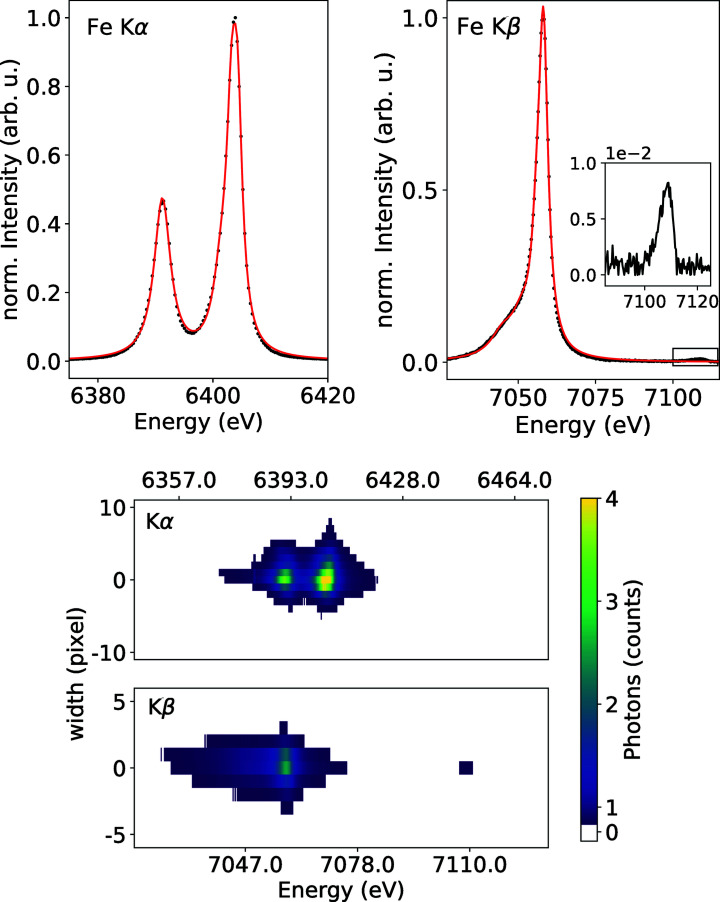
Upper figures: measured XES spectra from the Fe foil (black, dotted) for the *K*α (left) and *K*β lines including the background-corrected vtc signal (right), which were line-fitted using a combination of multiple overlapping Lorentzian peaks (red). Spectra including fitted Lorentzians for Ni, Co and Ge are shown in Figs. S3, S4 and S5. Lower figures: example of a 2D Jungfrau image showing mean photon counts per pulse reflected from an Fe foil and at full beamline transmission. Top: *K*α. Bottom: *K*β including vtc.

Spectra shown in Fig. 3 ▸ for Fe and Figs. S3, S4 and S5 for Ni, Co and Ge are each collected over 3000 trains with 10–200 pulses per train using all four crystals simultaneously with a maximum spectral window of Δ*E* = 150 eV. *K*β emission signals also include the relatively low intensity vtc signal at higher emission energies. Fe *K*α was measured using Si(333) and Ni and Ge *K*β using Si(444) reflections. Fe and Co *K*β as well as Ni and Co *K*α were measured with a set of Si(531) crystals. The separate spectra for each of the four crystals were added together after energy calibration. The resulting mean energy dispersion was calculated to be 0.32 ± 0.01 eV pixel^−1^. To determine broadening effects on the signal by various sources such as the focus size of the X-ray beam or jitter effects due to the focusing scheme, we fitted a combination of Lorentzian peaks to each fluorescence signal to obtain a near-perfect fit (see Fig. 3 ▸ for Fe *K*α and *K*β). The FWHM of each *K*α_1_, *K*α_2_ and *K*β line from Fe, Ni and Co was compared with the respective reference spectra (Hölzer *et al.*, 1997 ▸) (see Table 2 ▸). The calculated mean instrumental broadening from this data set assuming a Gaussian broadening function is 2.14 ± 0.6 eV. When compared with Fe *K*β measurements of later runs with either a SASE or monochromatic beam using beam stabilization and a different focusing scheme that aimed at reducing the horizontal beam jitter (see footnotes of Table 2 ▸), the experimental broadening was improved to 0.75 ± 0.07 eV which is far below the core hole lifetime broadening. Thus, the main fraction of the broadening can be traced back to an increase of the source size caused by spatial beam jitter and/or drift during the first experiment. We also determined the instrumental resolution using the elastic line measured with a monochromatic beam, which is in good agreement with the data presented from the emission experiments (see Section 5).

The integrated efficiency Ω of the spectrometer for an energy bandwidth FWHM of the rocking curve is calculated by the angular acceptance along the non-dispersive direction *l*_c_ multiplied with the integrated reflectivity *R*, the latter representing the area under the rocking curve of the crystal. The crystals with *l*_c_ = 110 mm are placed at a distance of 250 mm from the sample which corresponds to a horizontal angular acceptance of 433 mrad. The rocking curves for the most commonly used reflections Si(531), Si(333) and Si(444) were calculated for Bragg angles of 60°, 70° and 80° using the 1D Tagaki–Taupin equations (Takagi, 1962 ▸, 1969 ▸; Taupin, 1964 ▸) in the *pyTTE* package (Honkanen & Huotari, 2021 ▸) (see Fig. S6). For the calculations, we assumed a bending radius of 250 mm and a wafer thickness of 180 µm.

Based on data in Table 3 ▸ the spectrometer collects for Si(531), Si(333) and Si(444) between roughly 2.45 × 10^−5^ eV^−1^ and 12.90 × 10^−5^ eV^−1^ of all photons emitted in 4π from a point source.

## 4. XES from DACs

The combination of XES measurements from an X-ray heated DAC at HED was described in an earlier report (Kaa *et al.*, 2022 ▸). We followed the same strategy regarding setup geometry and sample loading while using the improved spectrometer with more analyser crystals and more degrees of freedom for the detector positioning, to achieve higher quality data.

### 4.1. Spin state change of (Fe_0.5_Mg_0.5_)O at 100 GPa

In the first of two test experiments (Konopkova & Sternemann, 2021 ▸), we used high repetition X-ray pulses (2.2 MHz and 50 pulses train^−1^) to heat (Fe_0.5_Mg_0.5_)O pressurized in a Ne pressure-transmitting medium to 100 GPa. The (Fe_0.5_Mg_0.5_)O powder was mixed with Pt which served as an *in situ* pressure standard and X-ray coupler for stronger X-ray absorption, which could also ensure indirect heating of the sample composed of lower-*Z* elements. The X-ray trains, arriving at 10 Hz repetition rate, induce a step-wise temperature increase over the duration of the X-ray trains while the sample partially cools down between the trains (Meza-Galvez *et al.*, 2020 ▸; Cerantola *et al.*, 2021 ▸). During the high-temperature state, XES and XRD signals are collected in a combined fashion to give information on the electronic spin-state change of Fe via the *K*β_1,3_ emission, using four Si(531) crystals and its crystalline structure, respectively. The heating was controlled by attenuating the photon flux and was roughly estimated by the equation of states for Ne and Pt in the simultaneously measured XRD data.

**Figure 4:**
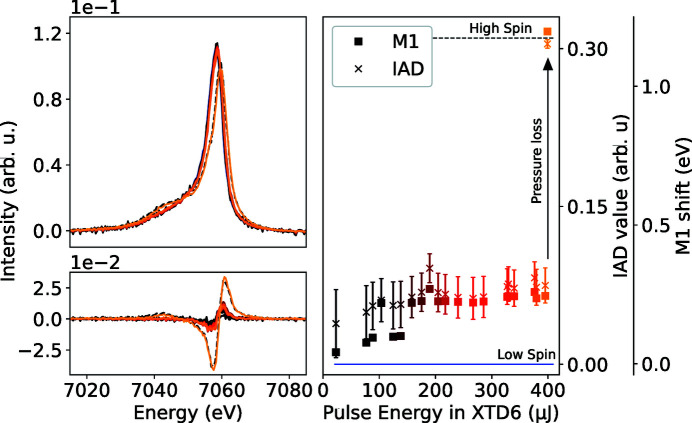
(Top, left) Fe *K*β emission for increasing pulse energies for (Fe_0.5_Mg_0.5_)O contained in a DAC at 100 GPa and after pressure loss. The difference from an LS reference (bottom, left) is slightly increasing with increasing pulse energies, hence implying an electronic spin-state increase. (Right) The IAD value (squares) and M1 shift (crosses) increase up to 200 µJ, after which it reaches a plateau. This would hint towards a value of average spin-state of *S* = 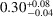 . The (Fe_0.5_Mg_0.5_)O (200) diffraction-peak shift (see Fig. S7) suggests a linear increase of the temperature with rising pulse energy. At the highest pulse energy, the pressure collapsed, and the spin-state switched into a full HS.

As suggested by earlier studies on (Fe_0.25_Mg_0.75_)O (Lin *et al.*, 2007 ▸; Mao *et al.*, 2011 ▸), ferropericlase undergoes a spin crossover from high spin (HS) to low spin (LS) state at 65 GPa and 300 K. At high pressures, in the LS state, it changes into a partial HS above 1300 K and back into a complete HS under pressure loss. With a higher fraction of Fe, we expect the pressure- and temperature-induced spin state to be changed at slightly higher pressures [see Liu *et al.* (2014 ▸) and references within]. In this experiment, we calculate the spin-state from the emission data by using the Integral of the Absolute Difference (IAD) value (Vankó *et al.*, 2006 ▸) relative to an LS and HS reference, and the first momentum (M1) shift of the *K*β main peak (Lafuerza *et al.*, 2020 ▸) relative to an LS reference. In a low spin state *S* = 0, the IAD and M1 shift values both correspond to a value of 0. When shifting to a high spin state *S* = 2, the IAD value reaches a value of 0.31 when the spectra are normalized to the integral in the energy range between 7020 and 7080 eV. The M1 shift reaches a value of 1.2 eV. The data presented in Fig. 4 ▸ show a step-wise increase in both values with increasing photon flux on the sample. The temperature increase based on the XRD data (see Fig. S7) suggests a linear increase with rising pulse energies. If we connect the increase in the IAD and M1 values to a change from LS state *S* = 0 to a higher spin-state, as suggested by, for example, Lin *et al.* (2007 ▸), the resulting spin-state value would be *S* = 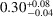 . At higher pulse energies, the IAD and M1 values exhibit a plateau. Upon further increase of the incident photon flux to 410 µJ, the upstream diamond fractured and caused a sudden drop in pressure that drove the transition of this possible medium spin-state to a complete high spin state.

### 4.2. Testing of pulse-resolved XES from a DAC

**Figure 5:**
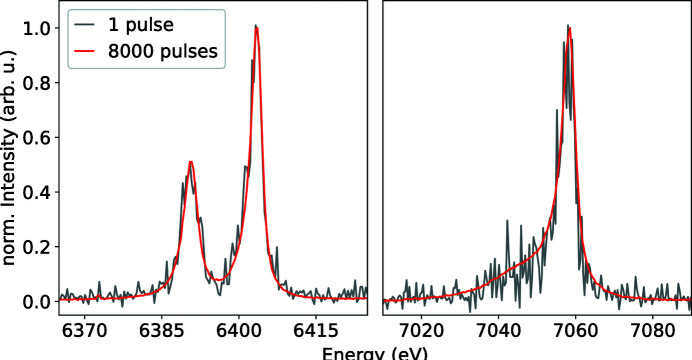
Emission from FeS at 32 GPa at single-bunch mode averaged over 8000 pulses (red). The *K*α emission (left) was measured using a single Si(111) analyser crystal, while the *K*β emission (right) was measured with three Si(531) analyser crystals simultaneously in a two-colour experiment. The accumulated signal shown for one crystal each over four pulses (grey) highlights the potential single pulse signal when measuring only one emission line with four analysers.

In a second DAC experiment (Cerantola & Sternemann, 2022 ▸) on FeS in Ne at 32 GPa, we tested the efficiency of Fe *K*β XES from a sample in a DAC using a single pulse of photons at full transmission. We used a combination of three Si(531) and one Si(111) analyser crystals to measure the Fe *K*β and *K*α fluorescence at the same time. The signal of one crystal each was accumulated over four pulses showing the highest intensity, to showcase the possible signal intensity of the *K*α and *K*β signal when using four crystals of the same type. For comparison, we accumulated the signal over 8000 trains also at full beamline transmission. Fig. 5 ▸ shows the difference in quality between the high-noise single pulse and the high-quality data integrated over 8000 pulses at 10 Hz (red). The *K*α emission, measured using the Si(111) crystal, shows a lower noise despite the higher photon absorption during the escape path through the upstream diamond at lower energies due to a roughly ten times higher fluorescence cross-section compared with the *K*β emission. Despite the high noise of the single-pulse *K*β emission signal the data quality is at a limit to detect large changes in spin-states. Future improvements in the DAC assembly such as an X-ray transparent gasket material or the use of mini-diamonds would increase the recorded signal in single-pulse-resolved MHz experiments. The use of X-ray transparent gaskets would allow for 90° measurements through the gasket, which would further improve the signal-to-noise due to lower X-ray scattering perpendicular to the horizontally polarized FEL beam. Furthermore, it could also decrease the amount of absorption of the fluorescence compared with exiting the DAC through the upstream diamond with an estimated travel length of 500 µm, by having a lower X-ray mass attenuation coefficient. Alternatively, as suggested by Lafuerza *et al.* (2020 ▸), the FWHM of the more intense *K*α, showing a sufficiently high signal-to-noise ratio in the single-pulse data, could be used to determine the electronic spin state. Such improvements could allow for time-resolved spin-state measurements from a DAC in future experiments. This could be done in combination with a femtosecond short optical pump laser and a single X-ray pulse-probe at up to picosecond time resolution as a function of the probe-delay, which would allow ultrafast electronic processes in statically pre-compressed samples to be studied. Depending on the stability of the sample environment and reversibility of the induced changes, several shots can be accumulated in a 10 Hz manner. Time-resolved measurements can also be performed using a MHz-resolved detector such as the Gotthard-II (Zhang *et al.*, 2021 ▸) in air or low vacuum, to resolve pulses within a pulse train at nanosecond to microsecond time resolution to study non-reversable heating induced changes in the electronic state of a sample. The latter is routinely done for measuring MHz resolved XRD from a DAC at HED (Liermann *et al.*, 2021 ▸).

## 5. IXS from ambient samples

To test the capabilities of the spectrometer for NRIXS measurements (Appel & Zastrau, 2022 ▸), the setup, using four Si(531) crystals, was placed in a forward-scattering direction at a scattering angle of roughly 20°. For these measurements and to improve the instrumental broadening, we used the first Si(111) double-crystal monochromator of the four-bounce monochromator setup at HED (Zastrau *et al.*, 2021 ▸), which was set to a photon energy of 7100 eV, with an energy bandwidth of Δ*E*/*E* ≃ 1.3 × 10^−4^. The SASE beam provided 2.2 mJ using 5 pulses train^−1^ at 455 kHz. It has to be noted that, due to the heating and subsequent expansion of the first monochromator crystal, the intensity of the later photon pulses within each train on the sample decreased significantly. For the energy calibration and additional XES measurements with a monochromatic beam, we measured the *K*β emission on a 5 µm Fe foil. The NRIXS measurements were tested by collecting the energy-loss spectra for plasmon and particle-hole excitation between 0 and 100 eV on a diamond plate, consisting of multiple crystals with different orientations along the measurement axis, with a thickness of 400 µm.

**Figure 6:**
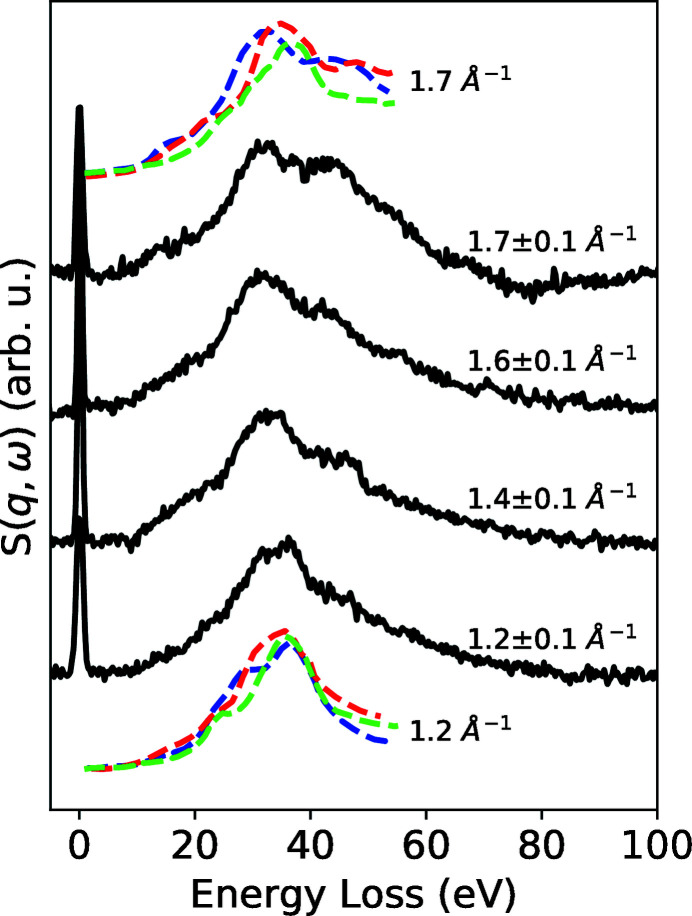
S(*q*, ω) of diamond for the experimental setup in forward scattering for crystals 1–4 utilizing different momentum transfers. Spectra measured by electron energy-loss spectroscopy along (110) (dashed, red), (111) (dashed, green) and (100) (dashed, blue) measured by Waidmann *et al.* (2000 ▸) are plotted for a momentum transfer of 1.7 Å^−1^ and 1.2 Å^−1^.

The position of the elastic line and the Fe *K*β signal compared with a reference was used to calibrate the energy axis. We fitted a combination of multiple Lorentzian peaks to the Fe *K*β signal. The FWHM of the *K*β signal was 3.62 eV due to the use of beam pointing stabilization. The elastic line (see Fig. S8) had a FWHM of 1.02 ± 0.23 eV contributed by the monochromator bandwidth of roughly 0.7 eV (Wollenweber *et al.*, 2021 ▸), thus resulting in a mean spectrometer bandwidth of 0.74 eV. The IXS signal was accumulated over 42000 trains. The signal was normalized based on the online intensity monitor using a photo-diode located upstream of the sample, in the optics hutch. To improve the signal-to-noise ratio, the trains with an intensity below a threshold intensity on the photodiode were filtered out. The signal, including the quasi-elastic line at 7100 eV, is shown in Fig. 6 ▸. We analysed the signal of each crystal with a spatial offset on the detector chip due to the different scattering angles φ, calculated from the horizontal and vertical scattering angle, of each of the four analyser crystals. Thus, each crystal provides measurement of the dynamic structure factor *S*(*q*, ω) of different wavevector transfer *q*. With increasing ω, *q* the measured signal shows a position shift, broadening and splitting of the main peak at 20–50 eV.

For comparison, we plotted reference IXS signals from a (110), (111) and (100) oriented diamond with a momentum transfer of *q* = 1.7 Å^−1^ and 1.2 Å^−1^ measured by Waidmann *et al.* (2000 ▸) using electron energy-loss spectroscopy, which is in relatively good agreement with the signal measured in our experiment. However, it has to be noted that the diamond sample is polycrystalline and the scattering signal originates from a combination of multiple unknown orientations. The bulk plasmon peak of diamond sits at 33 eV (Waidmann *et al.*, 2000 ▸; Vlasov *et al.*, 2012 ▸). With higher momentum transfer, the influence of interband transitions with similar energies increases. The resulting peaks shift to either higher or lower energies based on *q* and the crystal direction (Waidmann *et al.*, 2000 ▸). The signal shown in Fig. 6 ▸ could be interpreted as a convolution of scattering signal along multiple directions. This example demonstrates the capabilities of this setup to study electron–hole and plasmon excitation with eV-resolution.

Core electron excitation detected via XRS (Sternemann & Wilke, 2016 ▸) was tested with the same setup. Instead of adjusting the setup, we set the photon energy to 7380 eV to measure in the energy-loss range of 230–330 eV. The energy-loss spectrum on the diamond *K*-edge is shown in Fig. S9. The signal was accumulated over 45000 trains and filtered by the intensity in the same manner as mentioned before, resulting in a total accumulated train count of 30000. While the *K*-edge features are visible, the efficiency of the setup reaches its limits when using a quasi-single pulse pattern and the monochromator. XRS will become possible with seeded beam or higher monochromator throughput.

## 6. Conclusion

We successfully commissioned a von Hámos spectrometer that provides a setup to measure spectra in the hard X-ray regime from various samples contained in a DAC. The setup was tested in an energy range between 6400 and 11200 eV using Si(111) and Si(531) crystal cuts. It provides a sub-eV energy resolution with an energy window between 100 and 150 eV. It was successfully applied to fluorescence and electronic spin-state measurements from samples at high pressures and temperatures with SASE beam and IXS measurements using a monochromatic beam from ambient samples. Furthermore, the setup shows a sufficiently high efficiency for single-pulse XES measurements that ultimately enables MHz-resolved and pump–probe experiments from pre-compressed samples contained in a DAC. Thus this setup opens up a unique possibility to combine XRD and XES measurements from X-ray heated samples contained in a DAC at an XFEL beamline, which allows to systematically measure through the *pT* phase space of a sample at up to mbar pressure and potential melting temperatures. In the future it can be applied to measurements from samples in a DAC in combination with pump-and-probe laser heating and laser-driven shock compression.

## 7. Related literature

The following reference, not cited in the main body of the paper, has been cited in the supporting information: Verbeni *et al.* (2009 ▸).

## Supplementary Material

Supporting Figures S1 to S9. DOI: 10.1107/S1600577523003041/ok5088sup1.pdf

## Figures and Tables

**Table 1:** Available analyser crystal sets

Crystal	Orientation (*hkl*)	*d*-spacing (Å)
Si	531	0.9179
Si	111	3.1355
Si	533	0.8281
Si	511	1.0451
Ge	220	2.0000
Ge	111	3.2660

**Table 2:** FWHM of measured fluorescence signals (reference FWHM are given in parentheses)

	*K*α_1_	*K*α_2_	*K*β
Fe	3.28 (2.55)	3.59 (3.14)	4.58 | 3.62† | 3.60‡ (3.53)
Ni	2.83 (2.24)	3.69 (3.16)	5.99 (5.40)
Co	2.93 (2.33)	3.47 (3.18)	5.36 (4.37)
GeO_2_	–	–	8.14

† With monochromatic and highly stable beam (see Section 5).

‡ With SASE beam and highly stable beam (see Section 4).

**Table 3:** Peak reflectivity, FWHM and integrated reflectivity *R* for Si(531), Si(333) and Si(444) at Bragg angles of 60°, 70° and 80°, calculated from the rocking curves (see Fig. S6)

(*hkl*)	531	333	444
60°			
Peak reflectivity	0.60	0.73	0.67
FWHM (meV)	165.44	88.06	221.48
*R* (rad)	30.39	26.24	39.37

70°			
Peak reflectivity	0.58	0.71	0.66
FWHM (meV)	122.75	80.05	178.79
*R* (rad)	41.73	37.31	53.75

80°			
Peak reflectivity	0.57	0.70	0.65
FWHM (meV)	114.74	77.38	146.76
*R* (rad)	79.23	72.40	101.73
