# Supplementary material for: A von Hámos spectrometer for diamond anvil cell experiments at the High Energy Density Instrument of the European X-ray Free-Electron Laser
Source: J Synchrotron Radiat. 2023 May 9;30(Pt 4):822–30. doi: 10.1107/S1600577523003041 (PMC10325027; doi:10.1107/S1600577523003041)
Supplement: Supplementary file 1 [file s-30-00822-sup1.pdf]

JOURNAL OF SYNCHROTRON RADIATION

Volume 30 (2023)

Supporting information for article:

A von Hámos spectrometer for diamond anvil cell experiments at the High Energy Density Instrument of the European X-ray Free-Electron Laser

Johannes M. Kaa, Zuzana Konôpková, Thomas R. Preston, Valerio Cerantola, Christoph J. Sahle, Mirko Förster, Christian Albers, Lélia Libon, Robin Sakrowski, Lennart Wollenweber, Khachiwan Buakor, Anand Dwivedi, Mikhail Mishchenko, Motoaki Nakatsutsumi, Christian Plückthun, Jan-Patrick Schwinkendorf, Georg Spiekermann, Nicola Thiering, Sylvain Petitgirard, Metin Tolan, Max Wilke, Ulf Zastrau, Karen Appel and Christian Sternemann

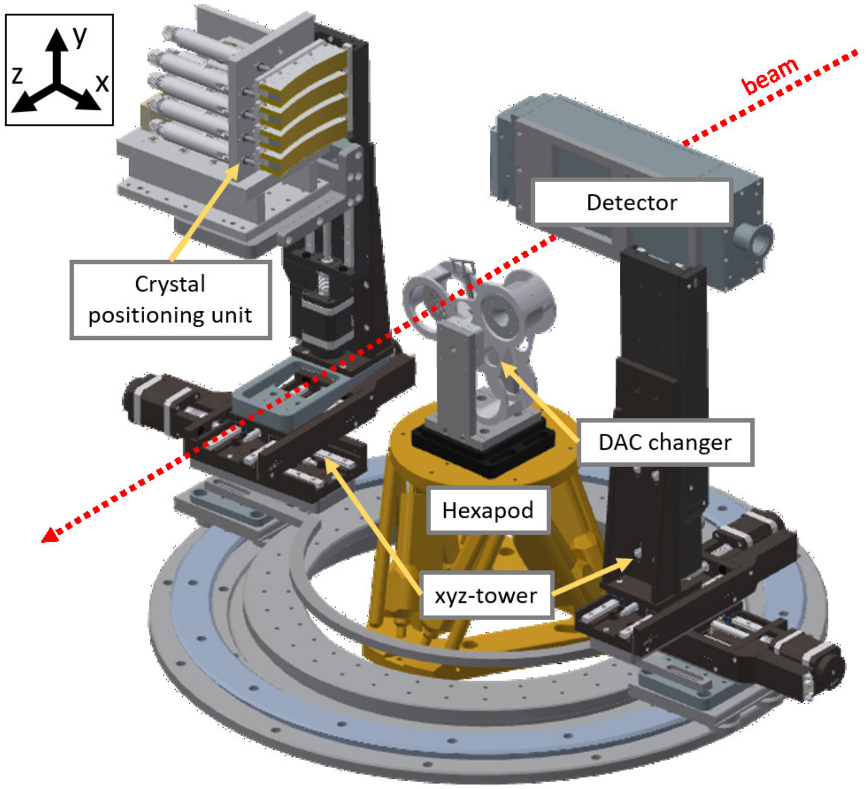

Fig. S1. CAD image of the spectrometer design in the vacuum chamber IC1 at HED. The spectrometer consists of two XYZ-towers located on a circular rail which are connected relative to each other on a fixed 180° angle. The four crystals are moved by a total of twelve linear motors (JJ X-Ray, 2021), four sets of three motors per crystal. The sensor of the 2D-Jungfrau detector is situated above the center of a hexapod, which is used for sample positioning.

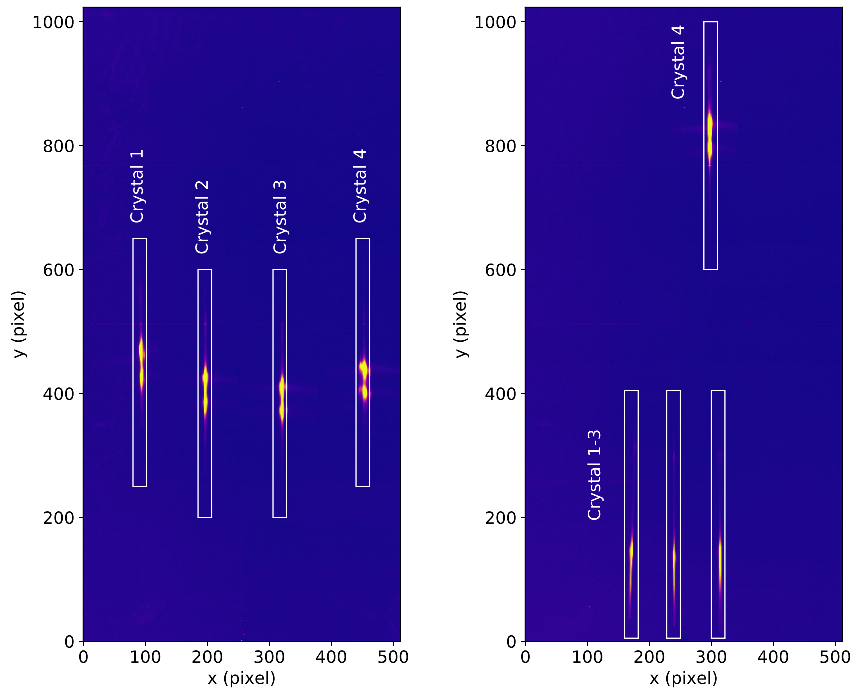

Fig. S2. [**Left**] Detector image for a Fe Kα measurement with four Si(111) crystals. [**Right**] Detector image for a Fe Kα and Kβ measurement with one Si(111) and three Si(531) crystals. The ROIs of each crystal are marked in white.

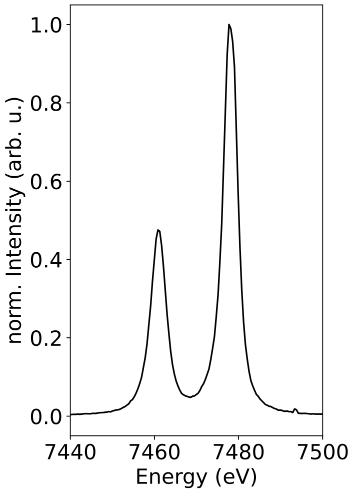

Fig. S3. **[Left]** Ni Kα emission **[Right]** Ni Kβ emission incl. vtc signal; measured

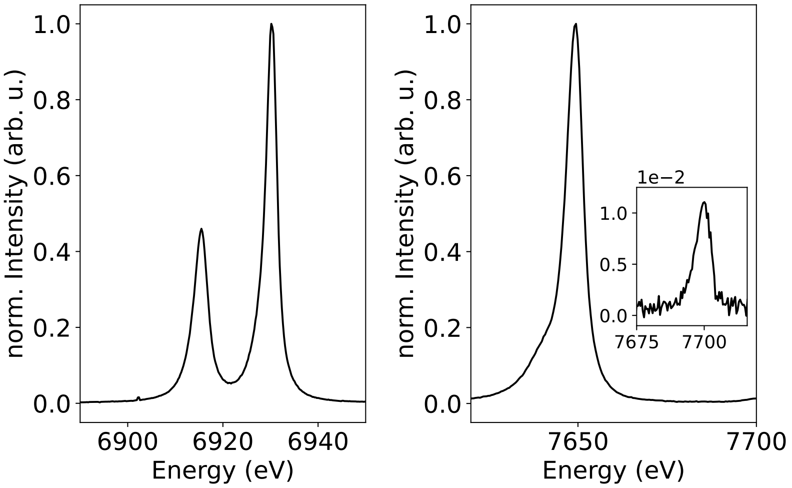

Fig. S4. **[Left]** Co Kα emission **[Right]** Co Kβ emission incl. vtc signal; measured

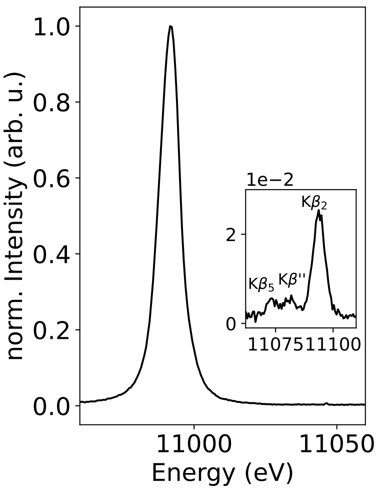

Fig. S5. GeO_2 Kβ emission incl. vtc signal; measured

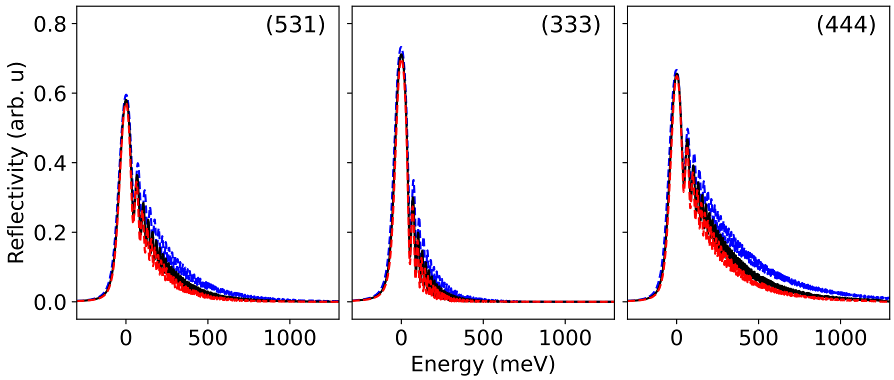

Fig. S6. Rocking curves for 250 mm bent, 180 µm thick Si(531), Si(333) and Si(444) crystals for a Bragg angle of 60° (blue, dashed), 70° (black, solid) and 80° (red, dashed) plotted against the energy difference to the Bragg energy. The data was calculated using the 1D Tagaki–Taupin equations (Takagi, 1962; Taupin, 1964; Takagi, 1969) in the pyTTE package (Honkanen & Huotari, 2021).

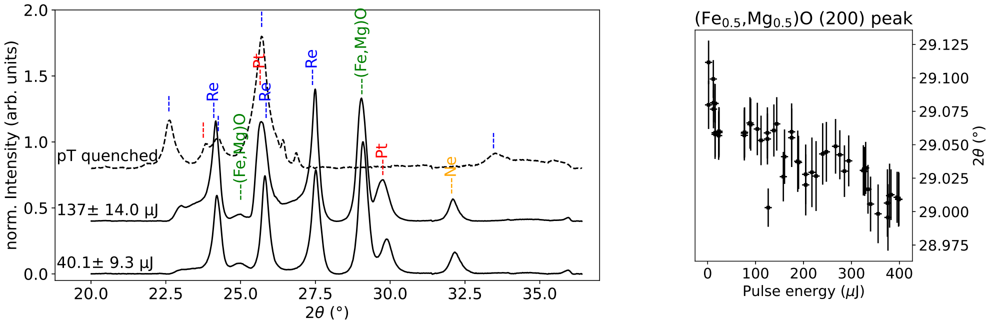

Fig. S7. [**Left**] XRD pattern taken during the experiment described in SUBSEC 4.1 for near ambient temperature runs and at 137 µJ. The pT-quenched state is shown as well (dashed). Re peaks (blue), Pt peaks (red), Ne peak (orange) and sample peaks (green) are marked. [**Right**] Peak shift of the $(Fe_{0.5}Mg_{0.5})O$ (200) peak with increasing pulse energies before the pressure collapse.

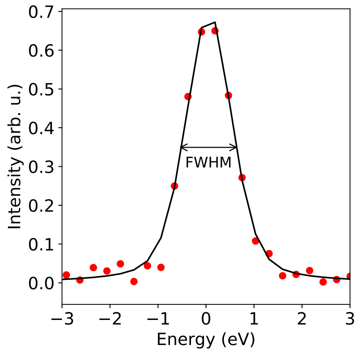

Fig. S8. Example for an elastic line for crystal 1 at 7150 eV using monochromatic beam. A Gaussian curve (black) is fitted to the measurements (red). The FWHM has a value of 1.1 eV.

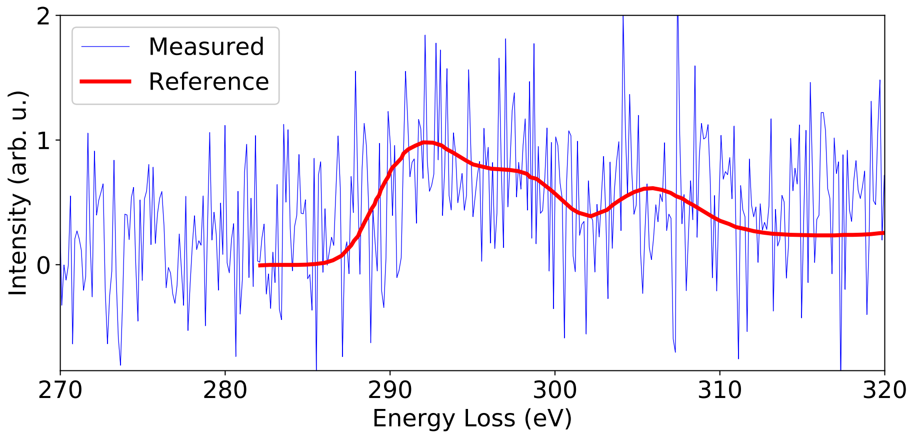

Fig. S9. Carbon K edge measured on a diamond plate background corrected (blue). The reference (red) was measured by Verbeni *et al.* (2009).
